# Hemodynamic and Metabolic Predictors of Unsuccessful Electrical Cardioversion or Early Atrial Fibrillation Recurrence After Electrical Cardioversion

**DOI:** 10.3390/jcm15197667

**Published:** 2026-10-03

**Authors:** Paulina Skalska, Małgorzata Kurpaska, Beata Uziębło-Życzkowska, Paweł Krzesiński

**Affiliations:** Department of Cardiology and Internal Medicine, Military Institute of Medicine National Research Institute, 04-141 Warsaw, Poland; mkurpaska@wim.mil.pl (M.K.); buzieblo-zyczkowska@wim.mil.pl (B.U.-Ż.); pkrzesinski@wim.mil.pl (P.K.)

**Keywords:** atrial fibrillation, atrial fibrillation recurrence, electrical cardioversion, hemodynamics, impedance cardiography, thoracic fluid content

## Abstract

**Background:** Rhythm control remains a cornerstone in the management of atrial fibrillation (AF). Although electrical cardioversion (ECV) effectively restores sinus rhythm (SR), its short-term durability is limited. **Aims**: This study aimed to evaluate the short-term rhythm outcomes after ECV and to identify predictors of failure to achieve or maintain SR within 30 days after ECV, with particular emphasis on the hemodynamic profile of patients with persistent AF. **Methods:** Ninety-four consecutive patients with persistent AF undergoing elective ECV were prospectively enrolled. Cardiac rhythm was assessed immediately after ECV, at ~24 h, and ~30 days. Laboratory testing, echocardiography, and impedance cardiography (ICG) were performed before ECV. Logistic regression analysis was used to identify predictors of failure to achieve or maintain SR within 30 days after ECV. **Results:** ECV was initially successful in 80 patients (85.1%), with SR maintained in 76 (80.8%) at 24 h. At 30 days, SR persisted in 49 patients (52.1%), while 43 patients (45.7%) failed to achieve or maintain SR, including 14 patients in whom ECV was unsuccessful and 29 patients who experienced AF recurrence after initially successful ECV; two patients were lost to follow-up. In multivariable analysis, T2DM was independently associated with higher odds of failure to achieve or maintain SR within 30 days after ECV (OR 3.26; 95% CI 1.183–8.978; *p* = 0.022). Higher TFC values were also independently associated with increased odds of this outcome (OR 1.15; 95% CI 1.017–1.291; *p* = 0.025). In contrast, the presence of hypertension was associated with lower odds of this outcome (OR 0.30; 95% CI 0.094–0.947; *p* = 0.040). Among hemodynamic parameters, only TFC was independently associated with the outcome, whereas echocardiographic parameters did not differ between groups. **Conclusions:** Even in a relatively low-risk population without advanced comorbidity burden, failure to achieve or maintain SR after ECV remains frequent. T2DM and elevated TFC were independently associated with failure to achieve or maintain SR, while hypertension was associated with a lower risk. These findings suggest that metabolic and hemodynamic abnormalities may provide prognostic information beyond that captured by conventional echocardiography.

## 1. Introduction

Rhythm-control strategies aimed at restoring and maintaining sinus rhythm (SR) using antiarrhythmic drugs or electrical cardioversion (ECV) remain one of the fundamental therapeutic approaches in the management of atrial fibrillation (AF). Restoration of SR improves cardiac hemodynamics by re-establishing atrial contraction and enhancing left ventricular (LV) filling and function [1]. These effects may alleviate symptoms, particularly in patients with suspected arrhythmia-induced cardiomyopathy, and reduce the risk of thromboembolic complications [1]. Despite the high immediate efficacy of ECV in restoring SR, its long-term effectiveness remains limited [2]. AF recurrence occurs in up to 71–84% of patients within the first year following the procedure [3]. Clinical factors associated with ECV failure and early (<30 days) AF recurrence include: AF duration, comorbidities (e.g., chronic kidney disease), female sex, and selected electrophysiological parameters [4]. However, these variables mainly reflect the final clinical manifestation of the disease rather than the underlying pathophysiological mechanisms predisposing to failure to achieve or maintain SR. Failure to maintain SR after ECV is believed to result primarily from persistent AF-related hemodynamic abnormalities, including LV volume overload and elevated left atrial pressure, leading to atrial remodeling and fibrosis that promote arrhythmogenicity. Conventional clinical and echocardiographic parameters may not fully capture these complex hemodynamic disturbances or the conditions associated with short-term rhythm failure [5]. Currently, noninvasive assessment of these mechanisms is feasible using echocardiography and impedance cardiography (ICG). Therefore, hemodynamic parameters, including indirect markers of volume and pressure overload, may play an important role in predicting ECV efficacy and the risk of AF recurrence. Accordingly, we aimed to assess the effectiveness of ECV during a 30-day follow-up and to identify factors associated with a primary composite endpoint comprising unsuccessful ECV or AF recurrence within 30 days after initially successful ECV, with particular emphasis on hemodynamic parameters assessed using echocardiography and ICG in patients with persistent AF.

## 2. Materials and Methods

### 2.1. Study Group

Patients with persistent AF who were admitted for elective ECV were recruited to the prospective study “Hemodynamic Profile of Patients with Persistent Atrial Fibrillation- Assessment of its Relation to Physical Efficiency and Effectiveness of Electrical Cardioversion as Through Hemodynamic Changes Related to Restoring Sinus Rhythm (HOPE-AF).” The recruited patients (January 2022–June 2023) were of both sexes, aged 18–80 years, with a ≤12-month history of persistent AF. All patients had a current AF episode lasting at least 7 days and therefore met the definition of persistent AF. Study exclusion criteria were arterial blood pressure at admission >180/100 mmHg, LV ejection fraction (LVEF) <40%, coronary artery disease, severe valvular disease, or history of surgery due to valvular dysfunction, history of cardiac pacemaker or other implantable device implantation, KDIGO category G4 or G5 chronic kidney disease, severe lung conditions (grade C or D chronic obstructive pulmonary disease, uncontrolled asthma, pulmonary arterial hypertension, pulmonary embolism), a severe systemic inflammatory disease, obesity with a body mass index (BMI) of >40 kg/m^2^, a severe mental disorder, a life expectancy of <12 months, musculoskeletal conditions significantly limiting mobility, and the absence of informed consent.

The study was conducted in accordance with the guidelines of Good Clinical Practice and the Declaration of Helsinki. The study protocol had been approved by a local ethics committee (30/WIM/2019, 19 June 2019), and each study subject gave their written informed consent.

### 2.2. Clinical Assessment

Each patient underwent a comprehensive clinical evaluation, including assessment of comorbidities, medication history, symptom severity using the European Heart Rhythm Association (EHRA) scale, and symptom burden using a visual analog scale (VAS). Physical examination included measurement of heart rate (HR), systolic blood pressure (SBP), diastolic blood pressure (DBP), and anthropometric parameters, including BMI.

### 2.3. Laboratory Tests

Peripheral venous blood samples were collected twice, prior to ECV and 30–40 days (at 8:00–10:00 a.m.) after successful ECV (if SR persisted). The blood tests included a complete blood count and measured the levels of NT-proBNP, creatinine, sodium, potassium, urea, glucose, lipids and folic acid. The estimated glomerular filtration rate (eGFR) was calculated using the Modification of Diet in Renal Disease (MDRD) equation. Hyperuricemia was defined as serum uric acid > 7 mg/dL in men and >6 mg/dL in women [6], and anemia was defined as hemoglobin levels <13 g/dL in men and <12 g/dL in women [7]. Due to the effect of AF on serum NT-proBNP levels irrespective of heart failure, the adopted cutoff level for stratification purposes was >365 pg/mL [8].

### 2.4. Echocardiography

Echocardiographic examinations were performed using a Vivid E95 cardiovascular ultrasound system (General Electric, Horten, Norway). The assessed parameters included cardiac chamber dimensions, left ventricular (LV) wall thickness, LV systolic function, including left ventricular ejection fraction (LVEF) and two-dimensional speckle-tracking–derived global longitudinal strain (GLS), LV diastolic function assessed with tissue Doppler imaging (TDI), right ventricular systolic function, valvular structure and function, pulmonary artery dimensions, pulmonary valve acceleration time, and echocardiographic probability of pulmonary hypertension. Regional LV wall motion abnormalities were defined as hypokinesis involving at least two myocardial segments according to the standard 17-segment LV model. LV hypertrophy was defined as interventricular septal thickness ≥12 mm measured in the parasternal long-axis view. To facilitate interpretation, GLS values were reported as absolute positive values instead of their conventional negative representation.

### 2.5. Impedance Cardiography

ICG was performed with a Niccomo^TM^ device (Medis, Medizinische Messtechnik GmbH, Ilmenau, Germany) after 10 min of sitting rest. The quality of the ICG recordings was routinely assessed using the quality-control functions integrated into the NICCOMO system. Hemodynamic parameters were obtained on a beat-to-beat basis and averaged over a 30-s recording period. Recordings affected by evident artifacts or inadequate signal quality were excluded from further analysis. The same standardized measurement procedure was applied at all study time points. The final analysis included the following hemodynamic parameters: HR [bpm]; SBP [mmHg]; DBP [mmHg]; and thoracic fluid content (TFC) [1/kOhm], calculated as the reciprocal of base impedance: TFC = 1000/Z0; total artery compliance (TAC); stroke index (SI) [mL/m^2^], calculated with a Sramek–Bernstein equation for stroke volume (SV) and indexed by body surface area; cardiac index (CI) [(mL/min)/m^2^], calculated as the product of SI and HR; velocity index (VI), which shows peak aortic blood flow; acceleration index (ACI), which shows peak aortic blood flow acceleration; Heather index (HI), defined as the ratio of peak ventricular ejection flow to the time interval from the peak of the electrocardiographic Q-wave to the peak of the ICG wave; systemic vascular resistance index (SVRI) [(dynx s)/cm^5^/m^2^)], calculated as 80 × (mean blood pressure − central venous pressure)/CI, where central venous pressure is assumed to be 6 mmHg [9].

### 2.6. Electrical Cardioversion

ECV was performed in accordance with standard clinical guidelines. After giving their informed consent, the patients underwent the procedure in a monitored setting, with continuous electrocardiographic and vital signs monitoring. ECV was performed under brief intravenous anesthesia by means of a synchronized biphasic shock from a cardioverter/defibrillator, with a starting energy of 120 J; the energy was gradually increased up to 200 J if the shock was ineffective. Up to three cardioversion attempts with increasing energy were attempted.

### 2.7. Statistical Analysis

Statistical analysis was performed using Statistica software, version 12.0 (TIBCO Software Inc., Palo Alto, CA, USA). The normality of distribution for continuous variables was assessed using the Shapiro–Wilk test.

Continuous variables were presented as the mean (SD) for normally distributed data or as the median and interquartile range (IQR: Q1–Q3) for non-normally distributed data. Categorical variables were expressed as absolute values and percentages.

Comparisons of clinical and hemodynamic parameters between patients with maintained SR and those with the primary composite endpoint were performed using appropriate statistical tests. Continuous variables were compared using the Student’s *t*-test or the Mann–Whitney U test, depending on data distribution. Categorical variables were compared using the chi-square (χ^2^) test or Fisher’s exact test, as appropriate.

To identify factors associated with the primary composite endpoint, defined as unsuccessful ECV or AF recurrence within 30 days, univariate logistic regression analysis was performed. Variables with a *p*-value < 0.10 in univariate analysis were entered into the multivariable model. Multivariable logistic regression analysis was then performed to identify independent predictors of the primary composite endpoint. For binary variables, the absence of the condition was coded as 0 and used as the reference category, whereas the presence of the condition was coded as 1. Results were expressed as odds ratios (ORs) with 95% confidence intervals (CIs).

All statistical tests were two-tailed, and a *p*-value < 0.05 was considered statistically significant.

## 3. Results

### 3.1. Baseline Characteristics of the Study Population

A total of 94 patients were included in the study (mean age, 65.9 ± 9.3 years; 69% male). The most common comorbidities were HTN (79.8%), obesity (42.6%), heart failure with preserved or mildly reduced left ventricular ejection fraction (LVEF ≥ 40%; 28.7%), and type 2 diabetes mellitus (T2DM; 27.8%). The duration of the current AF episode prior to ECV was 6–12 months in 35.1% of patients (*n* = 33), 3–6 months in 31.9%, and <3 months in 28.8% (Supplementary Figure S1). Most patients had not undergone previous rhythm restoration attempts (69.1% had no history of previous ECV, and none had undergone pharmacological cardioversion), whereas 14.9% had a history of AF ablation. Amiodarone therapy prior to ECV was reported in 12.8% of patients and was the only antiarrhythmic therapy used. Baseline pharmacotherapy was generally similar between groups. The only significant difference was a higher use of beta-blockers in patients who remained in SR at 24 h (91.9% vs. 70.0%; *p* = 0.02), with no differences at 30 days or for other medications. No significant changes in antiarrhythmic or rate-control therapy were observed during the 30-day follow-up period. Pre-ECV echocardiography demonstrated LVEF < 50% in 34% of patients and LV hypertrophy in 21.3%.

Basic characteristics of the study group have been presented in Table 1 and Supplementary Table S1.

### 3.2. Effectiveness of ECV

**Figure 1:**
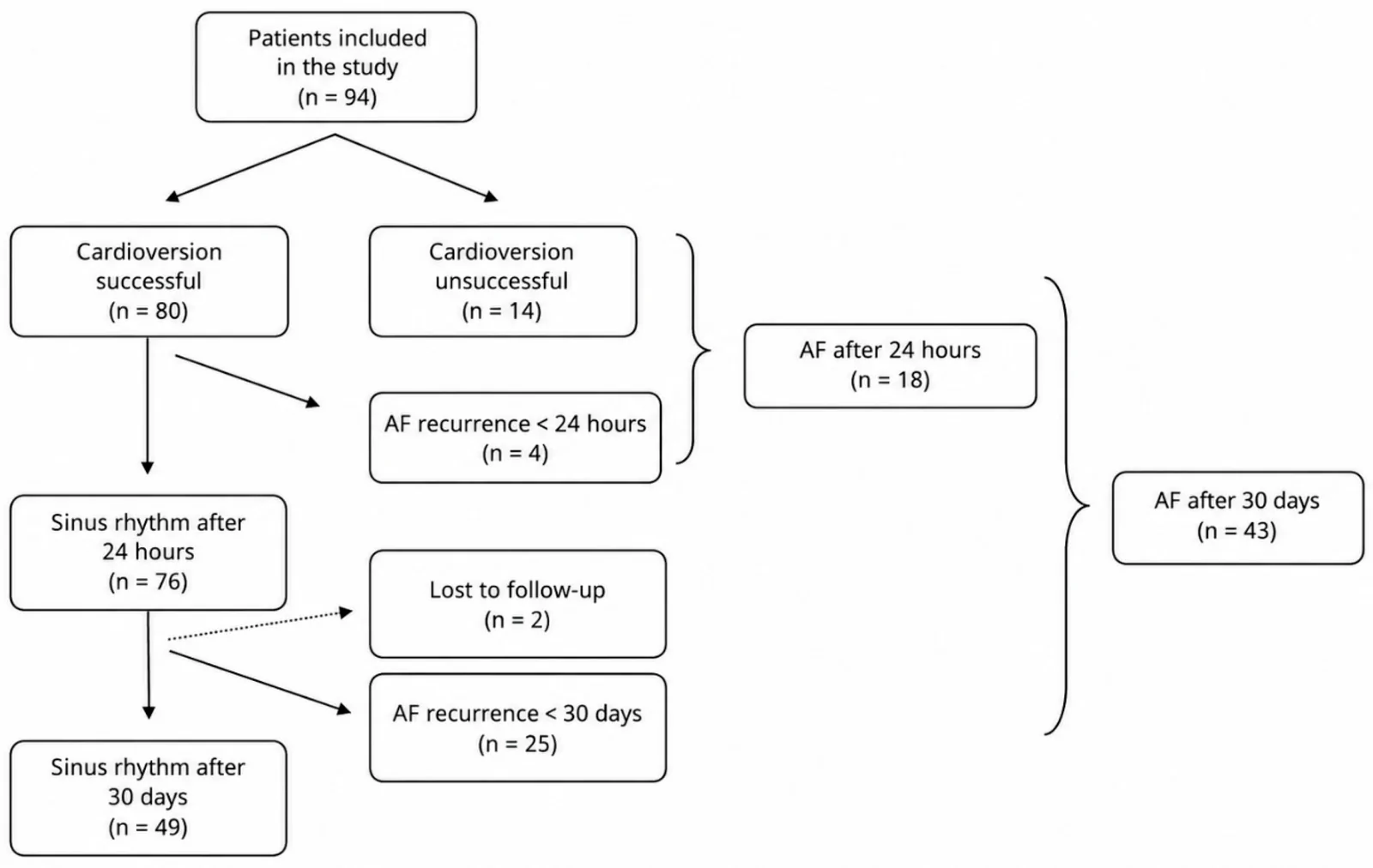
Patient flow according to sinus rhythm maintenance after electrical cardioversion. Solid arrows indicate the main study flow, whereas dotted arrows indicate patients lost to follow-up.

ECV was initially successful in 80 patients (85.1%). Early AF recurrence, defined as arrhythmia recurrence within 2 h after the procedure, was observed in 4 patients (4.3%). Thus, at 24 h after ECV, AF was present in 18 patients (19.1%), including 14 patients with unsuccessful initial ECV and 4 patients with early AF recurrence. During the remainder of the 30-day follow-up period, AF recurrence occurred in 25 of the 76 patients who were in SR at 24 h, while 2 patients were lost to follow-up. At 30 days, SR was maintained in 49 patients (52.1%). Overall, the primary composite endpoint, defined as unsuccessful ECV or AF recurrence within 30 days after initially successful ECV, was observed in 43 patients (45.7%). The proportion of patients maintaining SR over time is presented in Figure 1.

### 3.3. Comparison of Patients with Successful SR Maintenance vs. Those with Failure to Achieve or Maintain SR

Analysis of selected risk factors revealed significant differences between the groups with respect to the primary outcome following ECV. Compared with patients who achieved successful ECV and maintained SR during the 30-day follow-up, those who failed to achieve or maintain SR within 30 days after ECVhad lower rates of hypertension (HTN) (69.8% vs. 87.8%; *p* = 0.033), a trend toward a higher prevalence of T2DM (37.2% vs. 20.4%; *p* = 0.074), and higher thoracic fluid content (TFC) values (30.9 ± 4.1 vs. 29.1 ± 3.9 1/kOhm; *p* = 0.043).

No significant between-group differences were observed in echocardiographic parameters, including left atrial volume, moderate mitral regurgitation, or LV systolic and diastolic function. Similarly, none of the hemodynamic parameters assessed using ICG, except for TFC, differed significantly between groups. Moreover, there were no significant differences in the prevalence of comorbidities such as chronic obstructive pulmonary disease, chronic kidney disease, or obesity. The distribution of AF duration categories and the history of previous ECV/ablation were also comparable between groups. Likewise, no significant differences were observed in the use of diuretics or amiodarone, either at admission or at discharge. Baseline serum NT-proBNP and potassium levels were not significantly associated with the composite endpoint. Detailed data are presented in Table 2.

### 3.4. Predictors of Failure to Achieve or Maintain SR Within 30 Days After ECV: Univariate and Multivariate Analysis

In univariate analysis, the presence of HTN was associated with lower odds of failure to achieve or maintain SR within 30 days after ECV (OR 0.32; *p* = 0.039), whereas TFC values were associated with increased odds of the same composite endpoint (OR 1.12; *p* = 0.048). In addition, a trend toward higher odds of the composite endpoint was observed in patients with T2DM (OR 2.31; *p* = 0.078).

Variables with *p*-values <0.1 in univariate analysis were subsequently included in the multivariable model. In multivariable analysis, T2DM emerged as an independent predictor of failure to achieve or maintain SR within 30 days after ECV (OR 3.26; *p* = 0.022), despite showing only a trend toward significance in univariate analysis. Other independent predictors included higher TFC values (OR 1.15; *p* = 0.025), whereas HTN was independently associated with lower odds of the composite endpoint (OR 0.30; *p* = 0.040). The results of the logistic regression analysis of predictors of failure to achieve or maintain SR within 30 days after ECV are presented in Table 3.

## 4. Discussion

In the present study, failure to achieve or maintain SR after ECV was relatively common. Approximately 20% of patients experienced ECV failure or early AF recurrence within 24 h, while the primary composite outcome, defined as unsuccessful ECV or AF recurrence within 30 days, was observed in 43 patients (45.7%). Multivariable analysis identified T2DMand increased thoracic fluid content (TFC) as independent factors associated with the composite endpoint, whereas HTN was independently associated with lower odds of the composite endpoint. In contrast, no significant between-group differences were observed for the remaining echocardiographic or hemodynamic parameters.

Hypertension is a well-established risk factor for AF, increasing its incidence in the general population by approximately 40–50% [10]. However, its impact on the effectiveness of SR restoration and maintenance after ECV remains uncertain [11]. Some studies suggest that HTN promotes AF recurrence through enhanced atrial remodeling, including fibrosis and left atrial enlargement [12]. Moreover, a longer duration of HTN has been associated with an increased risk of early arrhythmia recurrence, indicating that the extent of hypertension-related structural remodeling may play an important role [13]. Conversely, other reports indicate that long-term antihypertensive therapy, particularly with angiotensin-converting enzyme inhibitors, angiotensin receptor blockers, and beta-blockers, may attenuate fibrotic remodeling and stabilize atrial electrophysiology, thereby facilitating SR maintenance after ECV [14]. The finding that a history of HTN was associated with lower odds of the composite endpoint in our study should therefore be interpreted with caution. Given the relatively small sample size and limited number of outcome events, this apparently paradoxical association may reflect residual confounding, model instability, or chance rather than a true protective effect of HTN itself. It is also possible that the observed association reflects specific characteristics of the study population rather than a direct biological effect of HTN. Future studies in larger cohorts should incorporate more detailed stratification to better characterize the interactions among HTN, comorbidities, hemodynamic profile, and AF recurrence risk. Potential confounding by concomitant pharmacotherapy and other clinical characteristics may also have contributed to this association.

Multivariable regression analysis identified T2DM as an independent predictor of the composite endpoint of unsuccessful ECV or AF recurrence within 30 days after ECV. Our findings are consistent with previous reports. Leopoulou et al. demonstrated that ECV efficacy in patients with T2DM was significantly lower than in individuals without diabetes, both immediately after the procedure and during 10–12 weeks of follow-up [15]. Patients with T2DM exhibit more advanced atrial remodeling [16]. In addition, cardiac autonomic neuropathy, a common complication of T2DM that may promote arrhythmia recurrence, appears to play an important role [16]. Furthermore, chronic inflammation, increased oxidative stress, endothelial dysfunction, and metabolic disturbances within cardiomyocytes may further exacerbate atrial remodeling. Collectively, these mechanisms contribute to the development of a complex arrhythmogenic substrate, including re-entry circuits responsible for AF maintenance and recurrence [17].

In light of previous reports, echocardiographic parameters reflecting left atrial morphology and LV systolic and diastolic function could be expected to differentiate patients according to their risk of AF recurrence after ECV. Numerous studies have demonstrated that greater left atrial volume, reflecting chronic pressure and volume overload, as well as reduced LVEF, are associated with an increased risk of arrhythmia recurrence [18,19]. However, in our study, standard echocardiographic parameters, including left atrial volume index (LAVI), LVEF, and indices of diastolic function, did not differ significantly between patients who maintained SR throughout the 30-day follow-up and those who failed to achieve or maintain SR within 30 days after ECV. This discrepancy may be partially explained by the relatively small sample size. Furthermore, the characteristics of the study population, including the relatively moderate severity of hemodynamic abnormalities, may have limited the magnitude of the observed differences. Importantly, commonly used echocardiographic parameters such as LAVI primarily reflect structural remodeling and may not fully capture microstructural alterations, including atrial fibrosis or electrophysiological abnormalities.

Our analysis also demonstrated no significant sex- or age-related differences in the composite endpoint, which is not entirely consistent with previous reports [11]. Although AF is more prevalent in men [20], some studies suggest that women are more susceptible to arrhythmia recurrence both after ECV and catheter ablation [21]. The lack of a similar association in our cohort may be related to the relatively short 30-day follow-up period. The effects of sex and age on AF recurrence risk are likely mediated largely through the extent of atrial remodeling [22], which is progressive over time. Consequently, the prognostic significance of atrial remodeling may become more apparent during longer-term follow-up.

The influence of additional comorbidities on the risk of AF recurrence after ECV has also been reported in previous studies. Conditions such as chronic kidney disease, obstructive sleep apnea, and chronic obstructive pulmonary disease have been shown to promote arrhythmia recurrence through mechanisms involving oxidative stress, chronic inflammation, autonomic dysregulation, and hypoxemia, thereby reducing the durability of ECV efficacy [5]. Obesity has likewise been identified as a major modifiable risk factor for AF recurrence [23], while higher CHA_2_DS_2_-VASc scores have been associated with impaired SR maintenance [24]. Our findings did not confirm these associations. The most likely explanation is the relatively low prevalence of some of these conditions within the study population, particularly chronic obstructive pulmonary disease and chronic kidney disease, which may have limited the statistical power to detect their effect on AF recurrence risk. Given the incompletely understood role of vitamin D in the pathophysiology of AF [25], further research is warranted to evaluate its potential association with ECV failure or early AF recurrence following a successful procedure. Among the hemodynamic parameters assessed using ICG, only TFC was significantly associated with the primary composite outcome. TFC reflects the total fluid content within the thorax [9] and may serve as an indirect marker of thoracic fluid status [26]. Importantly, NT-proBNP concentrations in the study population were elevated, although no patients presented with overt cardiac decompensation. Therefore, elevated TFC values may have reflected subtle and clinically silent alterations in thoracic fluid status. One possible explanation for the observed association is that increased TFC may be related to higher cardiac filling pressures, potentially resulting in atrial stretch and activation of mechanosensitive ion channels, which may promote shortening of the atrial refractory period and conduction heterogeneity [27]. These changes may contribute to an arrhythmogenic substrate that could predispose patients to AF recurrence even after initially successful ECV. Furthermore, increased atrial wall stress may promote fibroblast activation, contributing to fibrosis and electrophysiological remodeling of atrial tissue. Simultaneously, activation of neurohormonal pathways, including the renin–angiotensin–aldosterone system, may further enhance structural remodeling and atrial electrical instability. These potential mechanisms remain hypothetical, as they were not directly assessed in the present observational study.

However, it should be emphasized that TFC should be regarded as an indirect marker of thoracic fluid status, and the present study was not designed to establish the physiological determinants of elevated TFC or to demonstrate a direct relationship between TFC and intravascular volume overload.

## 5. Conclusions

The effectiveness of ECV remains limited, with nearly half of patients experiencing either unsuccessful ECV or AF recurrence within 30 days. In multivariable analysis, T2DM and elevated TFC were independently associated with higher odds of the primary composite endpoint, whereas HTN was associated with lower odds of this outcome. These findings suggest that the ability to achieve and maintain SR after ECV is associated with both metabolic and hemodynamic factors. Further studies are needed to determine whether these factors influence the ability to achieve and maintain SR after ECV and whether interventions targeting metabolic disturbances in T2DM or hemodynamic abnormalities reflected by TFC may improve post-ECV rhythm stability. Investigation in patients with more advanced hemodynamic impairment may further clarify these associations and their underlying mechanisms.

## 6. Limitations

The results of this study should be interpreted in light of several limitations. First, the relatively small sample size and single-center design may have limited statistical power and generalizability. Second, the short 30-day follow-up precludes assessment of long-term outcomes after ECV. AF recurrence was assessed using ECG recordings at predefined follow-up time points rather than continuous rhythm monitoring, which may have resulted in underdetection of asymptomatic or transient AF episodes occurring between assessments. Third, the primary endpoint combined primary ECV failure with AF recurrence after initially successful ECV, reflecting the overall short-term failure to achieve and maintain SR rather than a single pathophysiological mechanism. Although separate analyses of these clinically distinct scenarios would be informative, the relatively small number of patients and events precluded sufficiently powered subgroup analyses. Fourth, although the number of covariates in the final multivariable model was limited relative to the number of outcome events, the use of univariable *p*-value screening for variable selection may have introduced model instability and should be considered when interpreting the estimates.

In addition, the study population excluded patients with more advanced hemodynamic impairment and severe comorbidities, which may limit extrapolation of the findings to higher-risk populations. Hemodynamic parameters were assessed only once, prior to ECV, preventing evaluation of their dynamic changes over time. Finally, although multivariable analysis was performed, residual confounding, including differences in medical therapy and comorbidity control, cannot be excluded.

## Figures and Tables

**Table 1:** Baseline characteristics of the study group.

Variable	Study Group (n = 94) Mean ± SD/Median (Q1–Q3) or n (n%)
Age, years	68 (62–72)
Sex (females)	29 (30.9)
Body mass index, kg/m^2^	28.9 (26.4–31.4)
Heart failure	25 (26.6)
Hypertension	74 (78.7)
Previous stroke or transient ischemic attack	6 (6.4)
Type 2 diabetes mellitus	27 (28.7)
Chronic obstructive pulmonary disease	6 (6.4)
Peripheral artery disease	5 (5.3)
Chronic kidney disease	14 (14.9)
Sleepapnea	2 (3.1)
CHA_2_DS_2_-VASc (points)	3 (2–4)
Laboratory parameters	
Hemoglobin, g/dL	14.6 ± 1.2
Anemia	5 (5.3)
Sodium, mmol/L	141 (139–142)
Potassium, mmol/L	4.5 (4.2–4.6)
Creatinine, mg/dL	1.0 (0.9–1.1)
eGFR, mL/min/1.73 m^2^	90 (37–90)
eGFR < 90 mL/min/1.73 m^2^	19 (20.2)
Urea, mg/dL	38 (33–45)
Total cholesterol, mg/dL	164 (143–190)
HDL, mg/dL	55 (47–66)
LDL, mg/dL	92 (74–119)
Triglycerides, mg/dL	93 (75–122)
Fasting glucose, mg/dL	98 (92–109)
Uric acid, mg/dL	6 (5.2–7)
Hyperuricemia (>7 mg/dL M; >6 mg/dL F)	24 (25.5)
NT-proBNP, pg/mL	1021.0 (651–1352)
NT-proBNP ≥ 365 pg/mL	85 (90.4)
Impedance cardiography	
ICG systolic blood pressure, mmHg	129.7 ± 17.6
ICG diastolic blood pressure, mmHg	85.7 ± 11.1
ICG heart rate, bpm	89.3 ± 21.5
TFC, 1/kOhm	30.1 ± 4.1
CI, mL·m ^−2^·min^−1^	3.2 (2.8–3.8)
SVRI, dyn·s·cm^−5^·m^2^	2315 (1778–2766)
SV, mL	73 (64–95)
CO, L·min^−1^	6.3 (5.5–7.9)
VI, 1000·Ohm·s^−1^	32 (25–40)
ACI, 100·Ohm·s^−2^	50 (40–66)
HI, Ohm·s^2^	6.5 (4.7–8.8)
TAC, mL·mmHg^−1^	0.9 (0.8–1)
Echocardiography parameters	
Hypokinesis *	12 (12.7)
Moderate mitral insufficiency	9 (9.6)
Moderate tricuspid insufficiency	0 (0.0)
LAD, mm	46 (43–50)
RVDd, mm	30.7 ± 2.8
LVDd, mm	48.7 ± 4.6
Left ventricular hypertrophy	20 (21.3)
RAa, cm^2^	21.7 (19.0–24.5)
LAVI, mL/m^2^	46 (39.5–56.4)
LAVI > 34 mL/m^2^	81 (86.2)
GLS LV (%)	14.3 ± 2.8
Average E/e′	8.1 (7.3–9.9)
E/e′ ≥ 14	4 (4.2)
LVEF (%)	52.6 ± 6.8
LVEF 40–49%	32 (34)

Abbreviations: ACI, acceleration index; CHA_2_DS_2_-VASc (congestive heart failure/, left ventricular ejection fraction, LVEF ≤ 40%, hypertension, age ≥ 75 y, diabetes mellitus, stroke/transient ischemic attack/thromboembolism history, vascular disease; age 65–74; sex category); CI, cardiac index; CO, cardiac output; E/e′—ratio between early mitral inflow velocity and mitral annular early diastolic velocity; ECV, electrical cardioversion; eGFR, estimated glomerular filtration rate; GLS LV, global longitudinal strain of the left ventricle (GLS values are presented as absolute positive values); HAS-BLED (hypertension, abnormal liver/renal function, stroke, bleeding history or predisposition, labile INR, elderly, drugs/alcohol concomitantly); HDL, high-density lipoprotein; HI, Heather index; ICG, impedance cardiography; LAVI—left atrial volume index; LAD, left atrial diameter; LDL—low-density lipoprotein; LVDd, left ventricular diastolic diameter; NT-proBNP—N-terminal pro-B-type natriuretic peptide; SD standard deviation; RAa, right atrial area; RVDd, right ventricular diastolic diameter; SI, stroke index; SVRI, systemic vascular resistance index; TAC, total arterial compliance; TFC, thoracic fluid content; VI, velocity index; * hypokinesis of at least 2 segments.

**Table 2:** Comparison of baseline characteristics according to the primary composite endpoint.

Variable	SR Maintained at 30 Days After ECV (n = 49) Mean ± SD/Median (Q1–Q3) or n (n%)	Failure to Achieve or Maintain SR Within 30 Days After ECV (n = 43) Mean ± SD/Median (Q1–Q3) or n (n%)	*p*
Age, years	69 (63–70)	68 (62–75)	0.644
Sex (females)	14 (28.6)	15 (34.5)	0.516
Body mass index, kg/m^2^	29.7 (26.4–32.3)	28.7 (27.0–31.3)	0.467
Heart failure	13 (26.5)	11 (25.6)	0.978
Hypertension	43 (87.8)	30 (69.8)	0.033
Previous stroke/transient ischemic attack	4 (8.2)	1 (2.3)	0.218
Type 2 diabetes mellitus	10 (20.4)	16 (37.2)	0.074
Chronic obstructive pulmonary disease	3 (6.1)	3 (7.0)	0.868
Peripheral artery disease	3 (6.1)	2 (4.7)	0.756
Chronic kidney disease	9 (18.4)	5 (11.6)	0.369
Smoking	4 (9.1)	7 (17.1)	0.273
Anemia,	3 (6.1)	2 (4.7)	0.756
Hyperuricemia	13 (28.9)	11 (27.5)	0.887
Obesity	22 (44.1)	15 (34.9)	0.328
CHA_2_DS_2_-VASc (total)	3 (2–3)	3.0 (2–4)	0.454
Hemoglobin, g/dL	14.8 ± 1.1	14.6 ±1.4	0.356
Potassium, mmol/L	4.5 ± 0.4	4.46 ± 0.33	0.710
eGFR, mL/min/ 1.73 m^2^	90 (74.5–90)	90 (90–90)	0.332
NT-proBNP, pg/mL	1036 (596–1460)	1028 (713–1228)	0.993
Uric acid, mg/dL	6.2 (5.2–7.1)	6 (5.1–6.8)	0.503
Impedance cardiography			
ICG_systolic blood pressure, mmHg	128 ± 17	132 ± 18	0.346
ICG_diastolic blood pressure, mmHg	85 ± 10	86 ± 12	0.965
ICG_heart rate, bpm	92.1 ± 18	86.9 ± 25.1	0.070
TFC, 1/kOhm	29.1 ± 3.9	30.9 ± 4.1	0.043
SVRI, dyn·s·cm^−5^·m^2^,	2267 (1716–2688)	2353.5 (1872–2865)	0.360
SV, mL	72 (63–93)	74.5 (67–95)	0.824
CO, L·min^−1^	6.4 (6.0–8.1)	6.4 (5.4–7.7)	0.327
VI, 1000·Ohm·s^−1^	30 (24–38)	32.5 (25–41)	0.422
ACI, 100·Ohm·s^−2^	48 (40–64)	52 (36–71)	0.832
HI, Ohm·s^2^	6.2 (4.6–8.9)	6.7 (4.8–8.3)	0.878
TAC, mL·mm Hg^−1^	0.9 (0.7–1.1)	0.76 (0.76–0.97)	0.272
Echocardiography parameters			
Hypokinesis *	5 (10.4)	7 (17.1)	0.359
RVDd, mm	30.9 ± 2.8	30.7 ± 2.7	0.826
LVDd, mm	49.1 ± 5.1	48.3 ± 3.9	0.534
Left ventricular hypertrophy	13 (31)	7 (19.4)	0.246
Left ventricular mass index, g/m^2^	93 (69–106)	84 (75–105)	0.924
Raa, cm^2^	21.9 (19.5–24.4)	21.3 (18.4–24.8)	0.469
LAVI, mL/m^2^	46.3 (40.3–56.4)	47.4 (39.6–57.9)	0.880
LAVI > 34 mL/m^2^	42 (91.3)	37 (97.4)	0.242
GLS LV (%)	14.1 ± 2.8	14.5 ± 2.9	0.498
e′avg, cm/s	9.3 ± 1.9	9.52 ± 1.95	0.658
E/e′ avg	8.6 (7.6–9.9)	7.7 (6.6–9.8)	0.175
E/e′ > 14	2 (4.9)	2 (6.3)	0.798
TRPG, mmHg	25 ± 6.5	24.3 ± 7.0	0.358
EF (%)	51.4 ± 6.7	53.4 ± 6.8	0.107
LVEF < 50%	20 (41.7)	12 (29.3)	0.224

Abbreviations: see Table 1; e′, early diastolic mitral annular velocity; TRPG, tricuspid regurgitation pressure gradient. The primary composite endpoint was defined as failure to achieve or maintain SR within 30 days after ECV, including unsuccessful ECV and AF recurrence after initially successful ECV. Two patients were lost to follow-up and were therefore excluded from the 30-day outcome analysis. * hypokinesis of at least 2 segments.

**Table 3:** Logistic regression analysis of predictors of failure to achieve or maintain SR within 30 days after ECV.

Variable	Logistic Regression					
	Univariate			Multivariate		
	OR	95% CI	*p*	OR	95% CI	*p*
T2DM	2.311	0.912–5.858	0.078	3.258	1.183–8.978	0.022
HTN	0.322	0.110–0.942	0.039	0.298	0.094–0.947	0.04
TFC (per unit)	1.117	1.001–1.247	0.048	1.146	1.017–1.291	0.025
HR	0.989	0.969–1.008	0.254			

Abbreviations: T2DM—type 2 diabetes mellitus; HTN—hypertension; TFC—thoracic fluid content; HR—heart rate; OR—odds ratio; CI—confidence interval.

## Data Availability

The data presented in this study are available upon request from the corresponding author due to privacy restrictions.

## Supplementary Materials

The following supporting information can be downloaded at https://www.mdpi.com/article/10.3390/jcm15197667/s1: Figure S1: Distribution of study participants according to the duration of the current atrial fibrillation episode before electrical cardioversion; Figure S2: Distribution of atrial fibrillation duration categories according to 30-day rhythm outcome after electrical cardioversion; Figure S3: Distribution of patients according to previous atrial fibrillation ablation by 30-day rhythm outcome after electrical cardioversion; Figure S4: Distribution of patients according to previous electrical cardioversion by 30-day rhythm outcome after electrical cardioversion; Figure S5: Comparison of diuretic and amiodarone use at admission and at discharge according to 30-day rhythm outcome after electrical cardioversion; Table S1: Additional baseline characteristics of the study group.

## Abbreviations

The following abbreviations are used in this manuscript:

ACI	Acceleration time index
AF	Atrial fibrillation
BMI	Body mass index
CI	Cardiac index
CO	Cardiac output
DBP	Diastolic blood pressure
ECV	Electrical cardioversion
eGFR	Estimated glomerular filtration rate
EHRA	European Heart Rhythm Association
GLS	LV global longitudinal strain of the left ventricle
HI	Heather index
HR	Heart rate
HTN	Hypertension
ICG	Impedance cardiography
LAVI	Left atrial volume index
LV	Left ventricular
LVEF	Left ventricular ejection fraction
NT-proBNP	N-terminal pro-B-type natriuretic peptide
SBP	Systolic blood pressure
SI	Stroke index
SR	Sinus rhythm
SV	Stroke volume
SVRI	Systemic vascular resistance index
T2DM	Type 2 diabetes mellitus
TAC	Total arterial compliance
TFC	Thoracic fluid content
VI	Velocity index
