# Supplementary material for: Hemodynamic and Metabolic Predictors of Unsuccessful Electrical Cardioversion or Early Atrial Fibrillation Recurrence After Electrical Cardioversion
Source: J Clin Med. 2026 Oct 3;15(19):7667. doi: 10.3390/jcm15197667 (PMC13666183; doi:10.3390/jcm15197667)
Supplement: Supplementary file 1 [file jcm-15-07667-s001.zip › jcm-4485611-supplementary.pdf]

Supplementary Figures

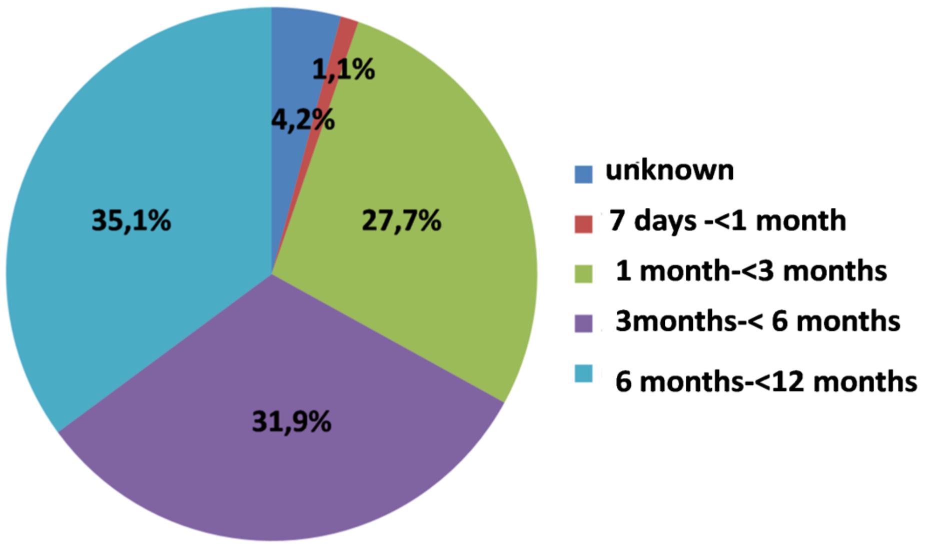

Figure S1. Distribution of study participants according to the duration of the current atrial fibrillation episode before electrical cardioversion.Values are presented as percentages of the study population.

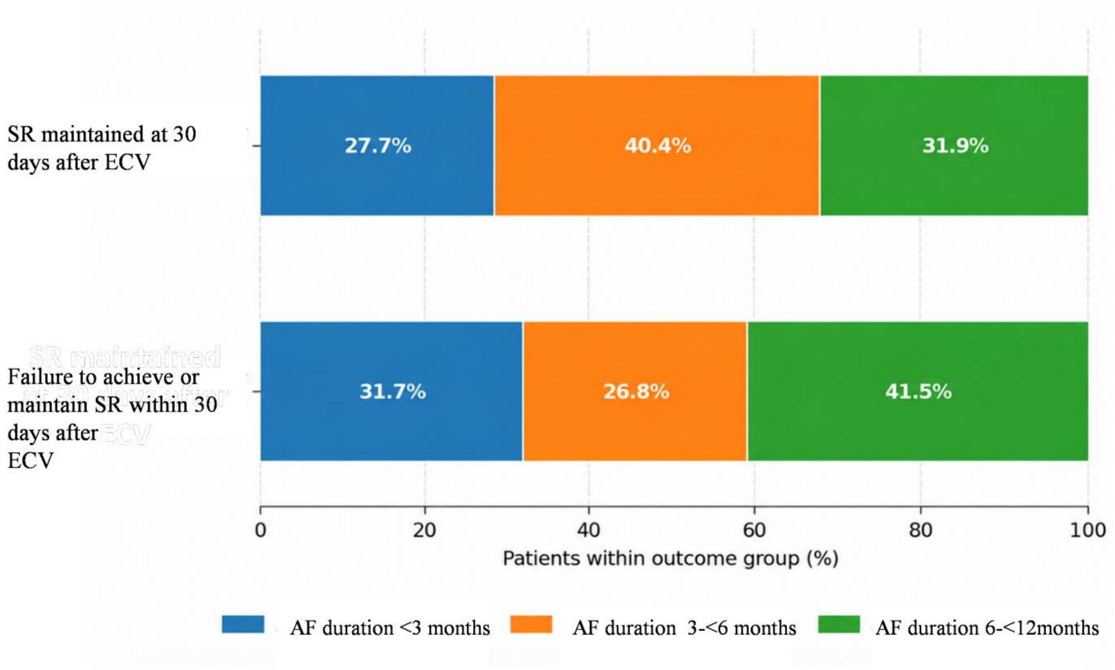

Figure S2. Distribution of atrial fibrillation duration categories according to 30-day rhythm outcome after electrical cardioversion (p = 0.395). Data are presented as percentages within the groups with maintained sinus rhythm and failure to achieve or maintain sinus rhythm at 30 days. Four patients had unknown AF duration, and two patients were lost to 30-day follow-up.

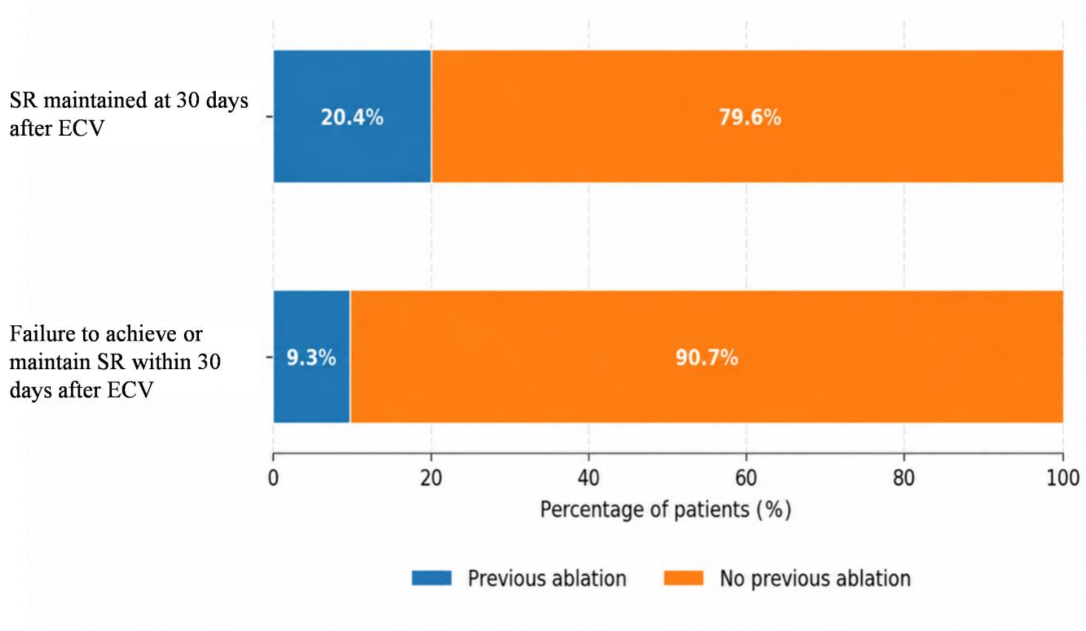

Figure S3. Distribution of patients according to previous atrial fibrillation ablation by 30-day rhythm outcome after electrical cardioversion ($p = 0.139$). Data are presented as percentages within the groups with maintained sinus rhythm and failure to achieve or maintain sinus rhythm at 30 days. Two patients were lost to 30-day follow-up.

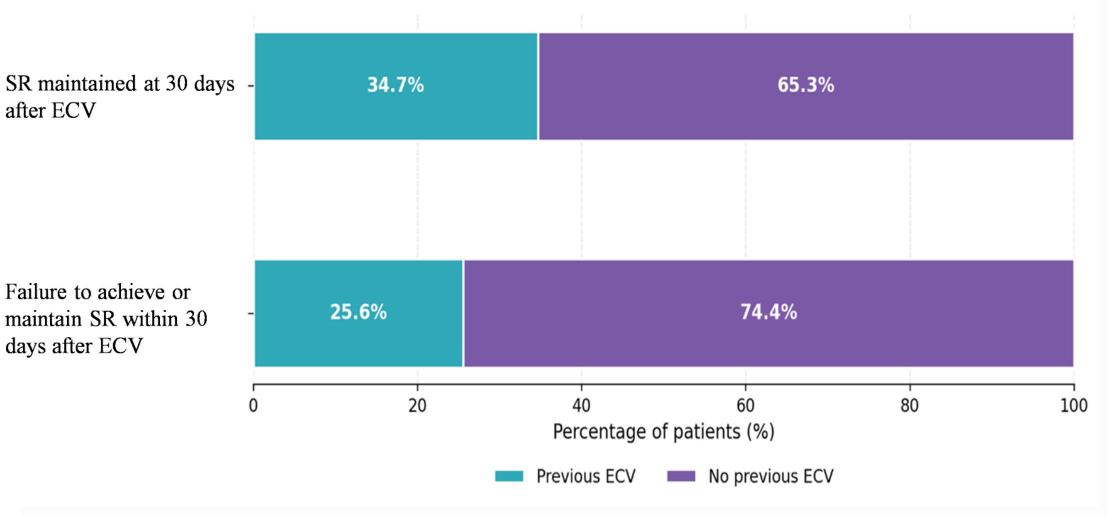

Figure S4. Distribution of patients according to previouselectricalcardioversion by 30-day rhythmoutcomeafterelectricalcardioversion (p = 0.343). Data are presented as percentages within the groups with maintained sinus rhythm and failure to achieve or maintain sinus rhythm at 30 days. Two patients were lost to 30-day follow-up.

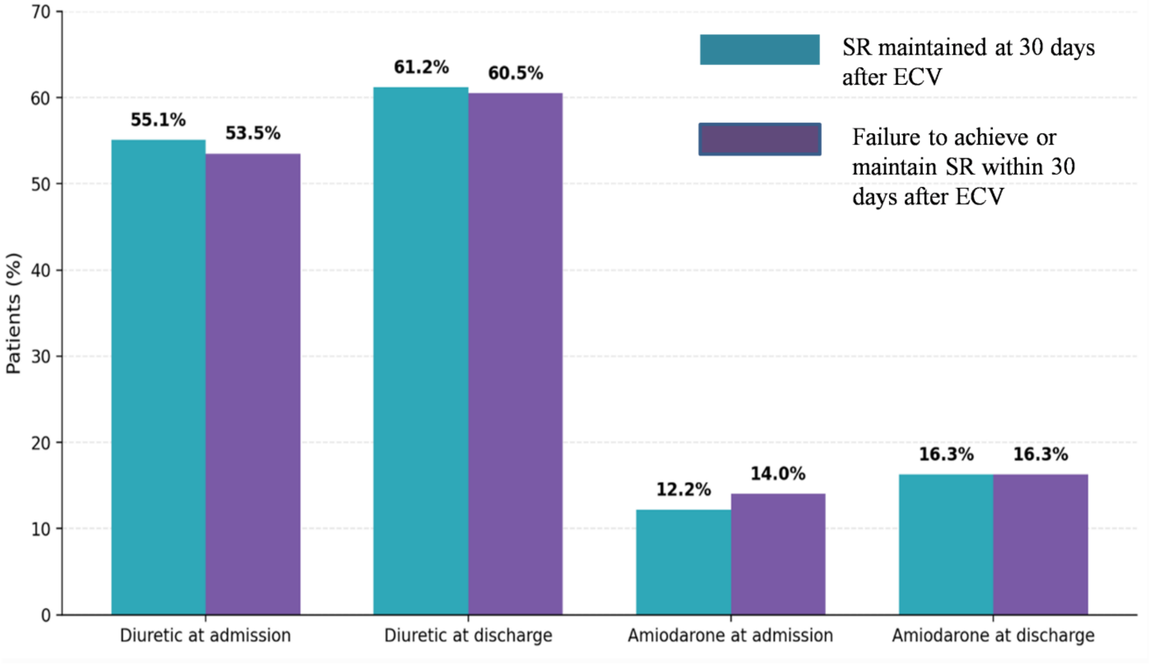

Figure S5. Comparison of diuretic and amiodarone use at admission and at dis charge according to 30-day rhythm outcome after electrical cardioversion (all p values non-significant). Data are presented as percentages within the groups with maintained sinus rhythm and failure to achieve or maintain sinus rhythm at 30 days.

Supplementary Table S1. Additional baseline characteristics of the study group

**Variable**	**Study group (*n* = 94)**<br>**n (%)**
Prior rhythm control interventions	
No previous ECV	65 (69.1)
Previous ECV <6 months	5 (5.3)
Previous ECV 6-12 months	9 (9.5)
Previous ECV >12 months	15 (15.9)
No previous FCV	94 (100)
Previous FCV <6 months	0
Previous FCV 6-12 months	0
Previous FCV >12 months	0
No previous AF ablation	80 (85.1)
Previous AF ablation <6 months	3 (3.2)
Previous AF ablation 6-12 months	5 (5.3)
Previous AF ablation >12 months	6 (6.4)
Smoking status	
Never smoker	64 (68.1)
Current smoker	12 (12.8)
Former smoker	18 (19.1)
Alcohol consumption	
Never	41 (43.6)
Former alcohol consumption	9 (9.6)
Occasional alcohol consumption	44 (46.8)
Daily alcohol consumption	0
Treatment at admission	
ACEi	48 (51.1)
ARB	18 (19.1)
Beta blocker	81 (86.2)
Calcium channel blocker	26 (27.7)

MRA	19 (20.2)
Diuretic	50 (53.2)
Amiodarone	12 (12.8)

Abbreviations: ACEi, angiotensin-converting enzyme inhibitors; ARB, angiotensin II receptor blockers; AF, atrial fibrillation; ECV, electrical cardioversion; FCV, pharmacological cardioversion; MRA, mineralocorticoid receptor antagonists.
